# Human placental villi contain stromal macrovesicles associated with networks of stellate cells

**DOI:** 10.1111/joa.13082

**Published:** 2019-09-11

**Authors:** E. Palaiologou, O. Etter, P. Goggin, D. S. Chatelet, D. A. Johnston, E. M. Lofthouse, R. Doherty, J. Pearson‐Farr, B. G. Sengers, C. Torrens, J. K. Cleal, A. M. Page, R. M. Lewis

**Affiliations:** ^1^ Human Development and Health Faculty of Medicine University of Southampton Southampton UK; ^2^ Biomedical Imaging Unit Faculty of Medicine University of Southampton Southampton UK; ^3^ Faculty of Engineering and Physical Sciences University of Southampton Southampton UK; ^4^ Institute for Life Sciences University of Southampton Southampton UK

**Keywords:** electron microscopy, extracellular vesicle

## Abstract

Placental function is essential for fetal development and establishing the foundations for lifelong health. The placental villous stroma is a connective tissue layer that supports the fetal capillaries and villous trophoblast. All the nutrients that cross the placenta must also cross the stroma, and yet little is known about this region. This study uses high‐resolution three‐dimensional imaging to explore the structural complexity of this region within the placental villi. Serial block‐face scanning electron microscopy and confocal microscopy were used to image the placental villous stroma in three‐dimensions. Transmission electron microscopy (TEM) was used to generate high resolution two‐dimensional images. Stereological approaches were used to quantify volumes of stromal constituents. Three‐dimensional imaging identified stromal extracellular vesicles, which constituted 3.9% of the villous stromal volume. These stromal extracellular vesicles were ovoid in shape, had a median length of 2750 nm (range 350–7730 nm) and TEM imaging confirmed that they were bounded by a lipid bilayer. Fifty‐nine per cent of extracellular vesicles were in contact with a fibroblast‐like stellate cell and these vesicles were significantly larger than those where no contact was observed. These stellate cells formed local networks with adherent junctions observed at contact points. This study demonstrates that the villous stroma contains extracellular macrovesicles which are considerably larger than any previously described in tissue or plasma. The size and abundance of these macrovesicles in the villous stroma highlight the diversity of extracellular vesicle biology and their roles within connective tissues.

## Graphical abstract

Extracellular vesicles are membrane‐bound structures released from cells into the plasma or stroma. Extracellular vesicles are thought to contain chemical messengers and have a signalling function. The two main classes of extracellular vesicle are exosomes and microvesicles, although because of their overlapping size distributions these cannot always be readily distinguished. Stromal extracellular vesicles are typically <300 nm in diameter but the placental villous stroma contains much larger ovoid stromal macrovesicles with a median length of 2750 nm. Villous stromal vesicles made up 3.9% of villous stromal volume.

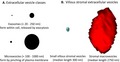

## Introduction

Placental function underpins fetal development and establishes the foundations for health in postnatal life (Burton & Fowden, 2015). All the nutrients and waste products that cross the placenta must cross the villous stroma between the maternal facing trophoblast and the fetal capillaries. However, relatively little research has focused on the villous stroma, its contents and their contribution to placental function.

Connective tissue is a fundamental component of all tissues and its maintenance and remodelling is essential for health (Bonnans et al. 2014). The connective tissue, or stroma, contains extracellular matrix and cells including fibroblasts and macrophages. Extracellular matrix consists of collagens surrounded by glycoproteins and proteoglycans which form a gel‐like material that determines many of the physical properties of the connective tissue (Yue, 2014). These extracellular matrix proteins are produced by fibroblasts, which also maintain and remodel the extracellular matrix.

Other components of the extracellular matrix are stromal extracellular vesicles (Rilla et al. 2017). These small membrane‐bound vesicles have been observed in a number of different tissues and are thought to contain mediators that help regulate stromal remodelling and processes such as angiogenesis (Rilla et al. 2017). In bone, extracellular vesicles of different sizes (from 20 to 300 nm) and different types have been reported in association with particular tissues or roles (Rilla et al. 2017). Extracellular vesicles can be made in a number of different ways; exosomes (20–250 nm) are thought to be made inside the cell and secreted whereas microvesicles (100–1000 nm) are thought to be made by folding and pinching off of the plasma membrane (Tong & Chamley, 2015).

In the placenta, extracellular matrix proteins are altered in pathological conditions such as maternal infection and idiopathic fetal growth restriction (Duaso et al. 2012). Within placental villi, the villous stroma forms a discrete layer of loose connective tissue underneath the syncytiotrophoblast and surrounding the fetal capillaries (Kaufmann et al. 2004).

The placenta is known to secrete extracellular vesicles into the maternal plasma and these are thought to carry bioactive signals which affect maternal physiology (Tong & Chamley, 2015). There is little known about stromal extracellular vesicles in the placenta but there is one report of large pericyte‐associated membrane‐bound structures in the villous stroma (Jones & Desoye, 2011).

Villous fibroblasts have a stellate structure with sparse perinuclear cytoplasm, and there is evidence that they produce the collagen and extracellular matrix proteins that maintain the structural architecture of the extracellular matrix (Ilic et al. 2008; Sati et al. 2008). Changes in extracellular matrix secretion have been reported when cultured placental fibroblasts are exposed to hypoxia, implicating fibroblasts in extracellular matrix‐related disease processes (Chen & Aplin, 2003). Fibroblasts and the extracellular matrix are also likely to regulate placental vascular development via fibroblast growth factors that stimulate angiogenesis (Auguste et al. 2003).

The role of the villous stroma in health and disease is poorly understood. This study uses three‐dimensional imaging at the nano‐ and micro‐scale to provide an insight into the role of elements of the stromal matrix, including placental fibroblasts and extracellular vesicles.

## Materials and methods

Term placental tissue was collected after delivery from uncomplicated pregnancies with written informed consent and ethical approval from the Southampton and Southwest Hampshire Local Ethics Committee (11/SC/0529).

### Tissue collection and fixation for electron microscopy

Villous samples from eight placentas were collected as soon as possible after delivery and small pieces (2 mm^3^) fixed in 3% glutaraldehyde in 0.1 m cacodylate buffer at pH 7.4 at room temperature (RT) and then stored at 4 °C for at least 24 h until processing for either serial block‐face scanning electron microscopy (SBF SEM) or TEM.

### TEM processing and imaging

Fixed placental fragments were washed twice for 10 min in 0.1 m sodium cacodylate buffer (Agar Scientific, UK) at pH 7.4 containing 0.23 m sucrose (BDH, UK) and 2 mm CaCl_2_ (BDH, UK). The specimens were then placed for 60 min in 2% osmium tetroxide (BDH, UK) in 0.1 m sodium cacodylate (Agar Scientific, UK) at pH 7.4, then washed three times for 10 min with distilled water. Samples were then treated with 2% aqueous uranyl acetate (Agar Scientific, UK) for 20 min. Samples were dehydrated using a graded ethanol series. Specimens were then treated with 50 : 50 Spurr resin : acetonitrile (Fisher, UK) overnight and infiltrated with fresh Spurr resin for 6 h. Finally, specimens were embedded in Spurr resin for 16 h at 60 °C.

Gold/silver ultrathin sections were cut using a Reichert Ultracut E ultramicrotome, stained with Reynolds lead citrate, and viewed by TEM to study structures associated with stromal vesicles and fibroblast‐like stellate cells (Tecnai 12, Thermo Fisher, Eindhoven).

### SBF SEM processing and imaging

Serial block‐face scanning electron microscopy is a high‐resolution technique where serial images are generated from a resin block which is sliced sequentially by an automated ultramicrotome in the chamber of a scanning electron microscope. Fixed samples for SBF SEM were processed based on Deerinck et al. (2010) as described in the Supporting Information Data S1. Briefly, samples were washed in 0.1 m sodium cacodylate buffer and immersed in 2% osmium and 1.5% potassium ferrocyanide in cacodylate buffer on ice for 60 min. Following this, samples were immersed in a 1% aqueous solution of thiocarbohydrazide (Acros Organics, UK) for 20 min and then in 2% OsO_4_ (Oxkem, UK) for 30 min, both at RT. This was followed by immersion in 2% aqueous uranyl acetate (Agar Scientific, UK) at 4 °C for 60 min, then in Walton's lead aspartate (Agar Scientific, UK) at 60 °C for 30 min. Between each step, samples were washed in distilled water. The samples were dehydrated through a graded ethanol series (Fisher, UK) and subsequently immersed in acetonitrile for 20 min and then 50 : 50 acetonitrile (Fisher, UK) and Spurr resin (Agar Scientific, UK) overnight. Samples were infiltrated with fresh Spurr resin for 6 h (Agar Scientific, UK), and embedded and polymerized at 60 °C for 16 h. Polymerized blocks were trimmed to < 1 mm^2^, mounted on an aluminium pin with conductive glue and sputter‐coated with gold palladium (Deerinck et al. 2010).

Blocks were imaged using a Gatan 3View (Gatan, Abingdon, UK) inside a FEI Quanta 250 FEGSEM (Thermo Fisher, Eindhoven, the Netherlands) at 3.0 kV accelerating voltage, spot size 3, and with a vacuum level of 40 Pa. Stacks of images were collected at pixel sizes ranging from 2.6 to 14 nm, slice thickness ranging from 25 to 50 nm, and number of slices was typically 300–2000. Only SBF SEM stacks that contained terminal villi were analysed in this study. The region of interest in the first slice was selected to include syncytiotrophoblast and stromal tissue.

### Wholemount confocal

To visualize networks of fibroblast‐like stellate cells, wholemount confocal microscopy was performed. Placental tissue was collected from normal pregnancies as described, and fixed overnight in 4% paraformaldehyde in phosphate‐buffered saline (PBS) pH7.4 (0.01 m phosphate buffer, 0.0027 m potassium chloride, 0.137 m sodium chloride). After overnight fixing, the specimens were washed in PBS. Samples were then stored at 4 °C.

Prior to labelling, the placental samples were again washed in PBS, cut into 2–3 mm^3^ blocks and permeabilized for 60 min in 1% Triton X‐100 (Sigma, UK) in PBS. The samples were then incubated in 2% bovine serum albumin (BSA, Fisher Scientific, UK) diluted in PBS to block non‐specific binding. Samples were then washed with PBS (3 × 10 min) and primary antibodies and/or lectins diluted in PBS were added to the samples and incubated overnight at 4 °C. Polyclonal rabbit anti‐human SLC22A11 (Abcam ab76385) at a dilution of 1 : 200 has been shown to bind to stellate cells in the villous stroma. Polyclonal mouse anti‐human CD163 (ABD Serotec MCA1853T) was used at 1 : 500. FITC‐Aleuria aurantia lectin (AAL, Vector Labs FL‐1391) was used at 1 : 150 dilution and rabbit anti‐human vimentin monoclonal (Clone SP20, Abcam ab16700) was used at 1 : 500. Samples were washed three times for 10 min, and incubated with secondary antibodies for 2 h on the roller with 0.4% 4’,6‐diamidine‐2’‐phenylindole dihydrochloride (DAPI; Sigma, UK) and then washed with PBS three times for 10 min. Secondary antibodies were supplied by Thermo Fisher and used at a dilution of 1 : 200.

Samples were cleared through an increasing series of thiodiethanol (TDE; Sigma, UK), 10% TDE in PBS, 25% TDE, 50% TDE, and finally three times in 97% TDE for a minimum of 30 min at each step. Samples were stored at 4 °C until imaging.

Samples were imaged using a 4‐channel confocal laser scanning microscope (SP5, Leica, UK). Stacks of 50–70 images were generated using sequential imaging (to eliminate spectral bleed through) with 800 × 800 pixels with 4‐line averaging (to reduce random noise) using ×63 objective magnification and ×3 optical zoom. Each stack consisted of between 50 and 70 sequential images with a Z‐axis spacing of 1 or 2 μm.

### Image processing and segmentation

Twenty‐seven SBF SEM image stacks of terminal villi from eight placentas were processed in fiji (version 2.0.0‐rc‐43) using a Gaussian blur filter (sigma radius 2) and enhance contrast (0.4% saturated pixels) (Schindelin et al. 2012). Selected regions were manually segmented in amira (Thermo Fisher, Eindhoven).

Confocal laser microscopy image stacks were also analysed using amira, to reconstruct in three‐dimensions the networks of fibroblast‐like stellate cells.

TEM images were all adjusted for brightness and contrast in photoshop.

### Quantification of volumes and diffusion barriers

To analyse volumes of villous components and the potential cellular barriers to diffusion in terminal villi, a stereological approach was adopted for SBF SEM stacks (Mayhew, 2006). It is important to note that these measurements are specifically for terminal villi, which are believed to be the primary exchange area within the villous tree. All the stereological analysis was performed by one person (E.P.). To calculate the volume of every cell type/area of interest in each stack, a random starting slice was selected from the first 50 slices and then every 50th slice until the end of the stack was analysed. A randomly offset 4 × 4 grid (giving 16 points where lines intersect) was applied to the selected slices using fiji (Gundersen et al. 1988). A total of 4944 points were categorized on 309 slices. This included 3136 points falling on villous components and 1808 on intervillous space.

To calculate proportional volumes of villous features, the number of points where the grid lines intersected falling in each cell type or area of interest (syncytiotrophoblast, cytotrophoblast, stroma, fibroblast‐like stellate cells, macrophages, pericytes, endothelial cells, endothelial lumen, and intervillous space) were counted and expressed as the proportion of the total number of points where the grid lines intersected falling on villous tissue (but not intervillous space). Whole tissue volume was determined from the number of points falling on the tissue as a proportion of total points falling on the image (including points not falling on tissue) multiplied by the volume of the region analysed.

To determine the length and width of the vesicles identified by the stereological approach, their locations were identified within the SBF SEM stacks and all the slices in which the vesicle appeared were inspected. The longest distance in the X‐, Y‐ and Z‐axes (as determined by block orientation) was measured. The longest axis was taken to be the length, and the average of the two shorter axes was taken to be the width.

### Quantification of typical‐size stromal vesicles

The volume of stromal vesicles of the typical sizes seen in other tissues (< 300 nm) was too low to allow smaller vesicles effectively to be identified by the unbiased systematic point counting approach. To estimate the size of these small vesicles, two to three vesicles per placenta were identified by eye and the length (longest axis) and width (average of the two shorter axis) were measured to provide an estimate of their size.

### Data analysis and statistics

For stereological analysis, running means were plotted to assess the adequacy of the sample size. Statistical comparisons were performed using a *t*‐test or Mann–Whitney *U*‐test as appropriate. A *P*‐value ≤ 0.05 was taken as significant. Data are presented as mean and SEM or median and range as appropriate.

## Results

This study included eight placentas, five collected following vaginal delivery and three following elective caesarean sections.

### Composition of the villus and villous stroma

The composition of the villous stroma in terminal villi was assessed in 27 SBF SEM stacks from eight placentas. The villous stroma was found to contain extracellular matrix, fibroblast‐like stellate cells, and extracellular vesicles (Table 1).

**Table 1:** Volume percentages of the components of terminal villi

	% of villous volume	% of stroma volume
	Mean ± SEM (*n* = 8 placentas)	
Stromal compartment	19.2 ± 3.4%	
Extracellular matrix	13.1 ± 2.2%	71.0 ± 4.7%
All extracellular vesicles	0.7 ± 0.2%	3.9 ± 1.3%
Fibroblast‐like stellate cells	4.6 ± 1.1%	21.6 ± 4.4%
Macrophages	0.8 ± 0.3%	3.5 ± 1.2%

### Extracellular vesicles

**Figure 1:**
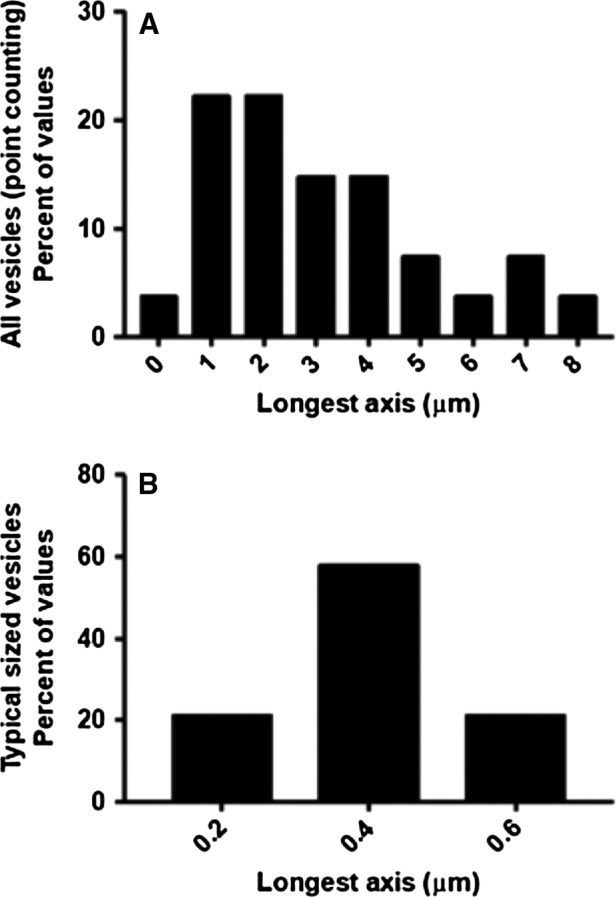
Size distributions of vesicles in the placental villous stroma. (A) All stromal vesicles identified by a non‐biased systematic point counting approach had a skewed distribution. (B) Typical‐size stromal vesicles identified by a non‐systematic approach targeting exosome‐like vesicles had a normal distribution.

Within the villous stroma, extracellular vesicles constitute 3.9% of stromal volume (Table 1). The length of the stromal vesicles (identified by systematic point counting) spanned a wide range and values were not normally distributed (Fig. 1). The stromal vesicles were ovoid with width‐to‐length ratios significantly lower than 1 (*P* < 0.001, Table 2).

**Table 2:** Dimensions of stromal extracellular vesicles

	Systematically selected stromal vesicles Median (range) (*n* = 27 vesicles, 8 placentas)	Typical‐size stromal vesicles Median (range) (*n* = 19 vesicles, 8 placentas)
Longest axis	2.75 (0.35–7.73) μm	0.30 (0.18–0.55) μm
Width	1.30 (0.20–6.22) μm	0.18 (0.12–0.28) μm
Width to length ratio	0.71 (0.25–0.96)	0.67 (0.25–0.82)

**Figure 2:**
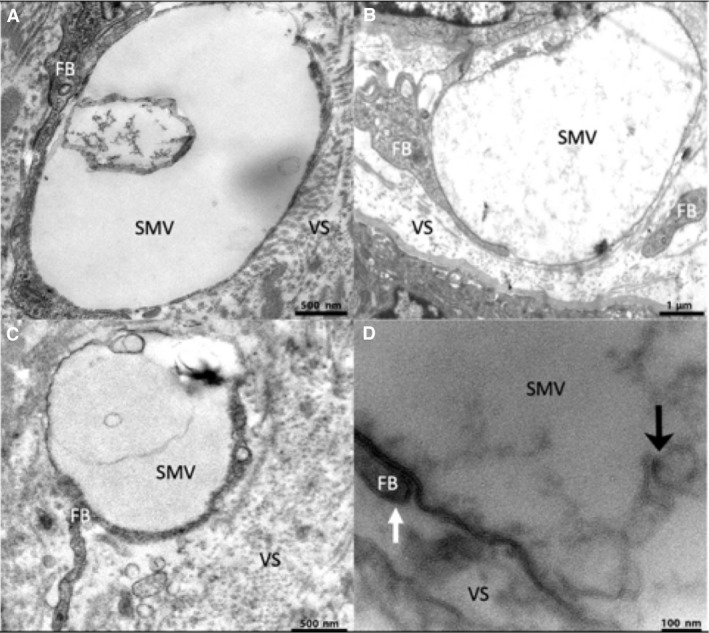
Transmission electron microscopy images of extracellular stromal macrovesicles showing contact with stellate processes and internal contents. (A) A stromal extracellular vesicle containing a membranous component in its cavity. (B) An extracellular vesicle containing diffuse material. (C) An extracellular vesicle containing a membranous component. (D) A higher magnification image showing the lipid bilayer of the vesicle membrane and an adjacent fibroblast process (white arrow) as well the membranous components (black arrow) inside the cavity of an extracellular stromal macrovesicle. FB, fibroblast‐like stellate cell; SMV, stromal macrovesicle; VS, villous stroma.

**Figure 3:**
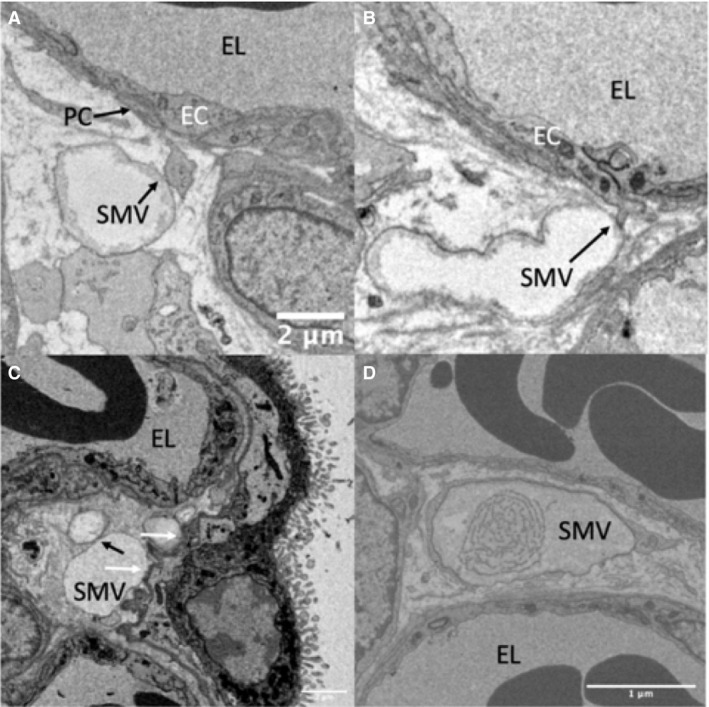
Serial block‐face scanning electron microscopy images of stromal macrovesicles making contact with structures other than stellate cells. (A) A pericyte process touching a stromal microvesicle. (B) An endothelial cell process touching a stromal macrovesicle (C) Shows stromal macrovesicles lying adjacent to the syncytiotrophoblast basal lamina (contact points indicated by a white arrow) and also adjacent to a separate vesicle (contact point indicated by a black arrow; these vesicles were shown to be distinct structures by inspecting serial sections). (D) An example of a vesicle whose contents had a higher degree of structure than was typically observed. EC, endothelial cell; PC, pericyte; SMV, macrovesicle.

Based on the 27 vesicles randomly selected by point counting, the extracellular vesicles were in contact with a stromal cell at some point on their surface in 63% of cases, with 16 in contact with fibroblast‐like stellate cells and one in contact with a macrophage. However, despite their close association with stromal cells the stromal macrovesicles appeared to be distinct structures with discrete membranes (Fig. 2). Vesicles in contact with stromal cells were significantly larger [median (range)] than those without [3.2 (0.7–7.7) μm, *n* = 18 vs. 1.65 (0.35–5.6) μm, *n* = 9, *P* = 0.025]. In a limited number of samples, TEM imaging of the stromal macrovesicle membranes demonstrated the leaflets of the lipid bilayer (Fig. 2D). The stromal extracellular vesicles were largely devoid of electron‐dense contents but they often contained strands of membrane or vesicles which in one case were demonstrated to be a lipid bilayer by TEM (Fig. 2D). However, the vesicles rarely contained more than one or two of these membranous structures and there was nothing resembling intact organelles. In isolated cases, there were instances of vesicles containing more structured contents, reminiscent of partially digested organelles, but this was not a consistent feature (Fig. 3D).

The majority of observed vesicle interactions were with fibroblast‐like stellate cells, but stromal vesicles were observed interacting with other structures including the trophoblast basal lamina, endothelial cells, and pericytes (Fig. 3). In a limited number of cases, contact between endothelial cells and pericytes was observed and in these cases rather than the two structures lying alongside each other it appeared that a cellular process from the endothelial cell or pericyte extended to touch the stromal vesicle (Fig. 3A,B).

As small stromal vesicles were not readily identified using the systematic approach described above, visual inspection of SBF SEM images was used to identify 19 typical‐size stromal extracellular vesicles from eight placentas. The dimensions of these vesicles are given in Table 1 and their lengths were found to be normally distributed (Fig. 1B). Extracellular vesicles within this size range represent 0.1% of stromal volume based on the systematic analysis above. The typical stromal vesicles were not observed to interact with the stellate cells, nor did they have visible contents. The vesicular nature of these structures was confirmed by scrolling through the SBF SEM stack.

Although this study is of SFB SEM stacks from terminal villi, large extracellular vesicles were also observed in SBF SEM stacks from intermediate villi. There was no apparent difference in the morphology of the extracellular vesicles in intermediate villi (data not shown).

### Fibroblast‐like stellate cells

**Figure 4:**
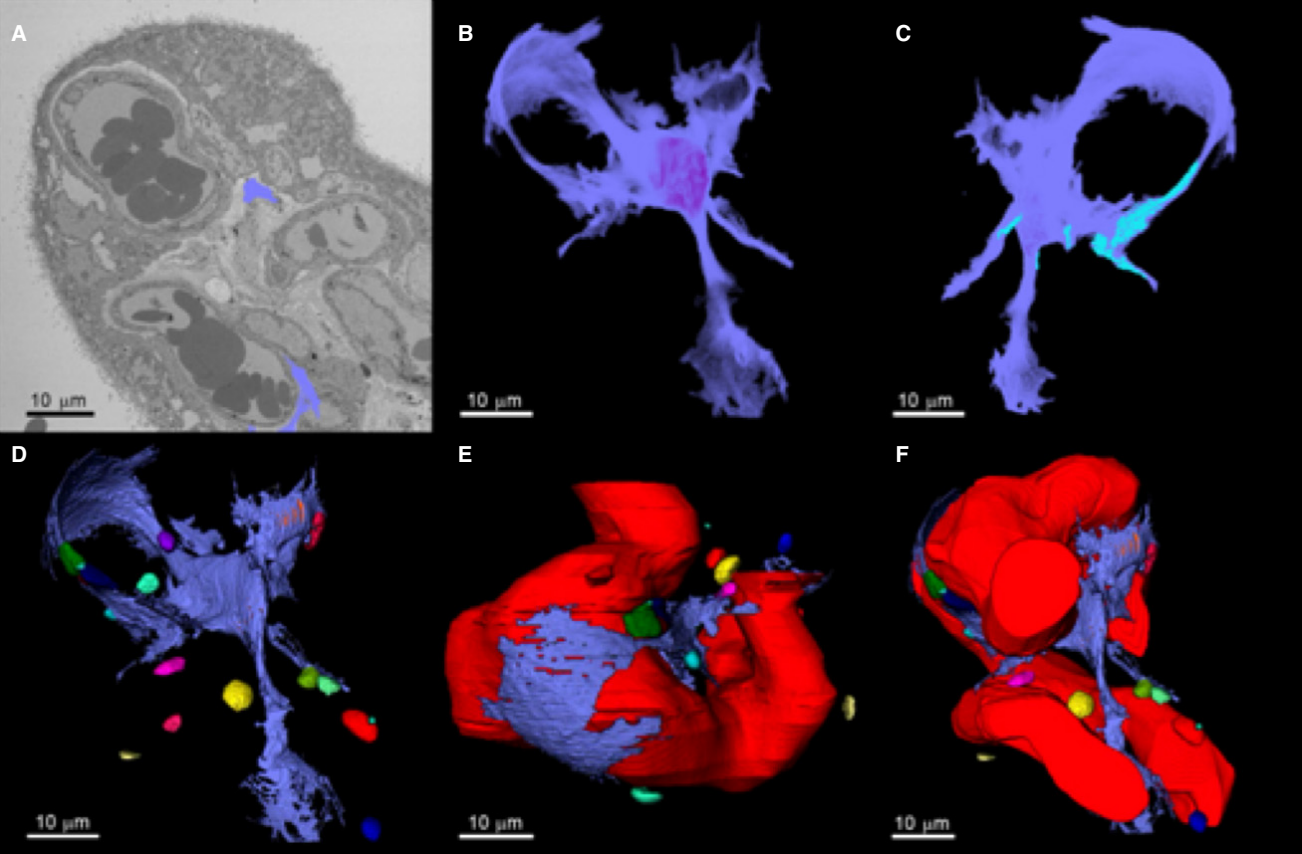
Three‐dimensional reconstruction of a fibroblast‐like stellate cell and its relationship with capillaries and stromal extracellular macrovesicles. (A) Serial block‐face scanning electron microscopy image of a placental villus showing the segmented processes of a fibroblast‐like stellate cell emerging from the slice (blue). Both of the blue segmented regions are part of the same cell. (B) Segmented three‐dimensional fibroblast structure. (C) Regions on the stellate cell where there were interactions with other stellate cells are shown in light blue. (D) Fibroblast interactions with extracellular macrovesicles in the villous stroma (see Fig. S1 for an interactive 3D model). (E) The relationship between the fetal capillary and the fibroblast. (F) Association of the stellate cell with the fetal capillary and the extracellular macrovesicles.

Within the three‐dimensional SBF SEM stacks, the fibroblast‐like stellate cells could be seen throughout the terminal villi, with many apparent cell‐cell connections. Segmentation and three‐dimensional reconstruction of a stromal stellate cell demonstrated a cell with limited perinuclear cytoplasm and multiple, long, thin processes extending out into the extracellular matrix (Fig. 4, an interactive 3D model is available in Supporting Information Fig. S1). Stellate processes were also in contact with extracellular stromal macrovesicles (Figs 2 and 4D). The processes from the fibroblast‐like stellate cells formed a thin sheet, or sail, and these were observed to be closely aligned with the underlying capillaries (Fig. 4E).

**Figure 5:**
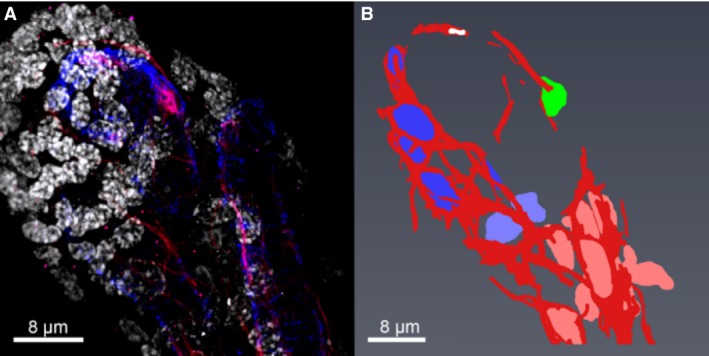
Wholemount confocal imaging of networks of fibroblast‐like stellate cells. (A) A projection of a confocal stack showing fibroblast‐like stellate cell networks stained with SLC22A11 (red), pericytes surrounding fetal capillaries stained with α‐SMA (blue), and the nuclei stained with DAPI (white). (B) Segmentation of the fibroblast‐like stellate cell networks shown in (A). The fibroblast‐like stellate cell processes are shown in red and their nuclei are shown in four different colours [blue, light blue, green (isolated cell) and pink], demonstrating three different networks.

A population of stellate cells in the stroma were shown to bind antibodies to SCL22A11 (Fig. 5) and vimentin (Supporting Information Fig. S2). The SCL22A11 antibody did not co‐localize with the macrophage marker CD163 (Fig. S2). Some SLC22A11 staining was also observed on discrete regions of endothelium, but this was easily distinguishable from stromal staining in a three‐dimensional stack using AAL, which bound most strongly to capillaries.

**Figure 6:**
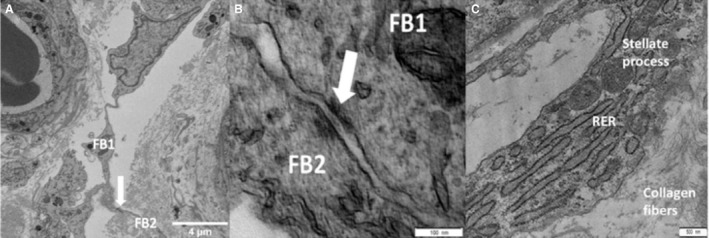
Electron microscopy images of placental stromal stellate cells. (A) A single serial block‐face scanning electron microscopy image showing the networks of stellate processes within the stroma with the white arrow indicating the junctional complex between two stellate cells (FB, fibroblast‐like stellate cell). (B) Transmission electron microscopy (TEM) image of two adjacent stellate cells connected with an adherens junction (white arrow). The two stellate cells are marked FB1 and FB2. (C) TEM image of a fibroblast process containing cellular machinery. RER, rough endoplasmic reticulum.

Confocal imaging showed fibroblast‐like stellate cells making connections with a number of fibroblast‐like stellate cells (Fig. 5). Analysis of fibroblast‐like stellate cell networks in confocal stacks from three placentas suggested that the average number of fibroblast nuclei per network was 3.3 ± 0.3 (although this may be limited by the size of the stacks). Figure 4F highlights the regions on the surface of the three‐dimensional fibroblast‐like stellate cell reconstruction, where adjacent cells were in contact with its surface (Fig. 4F). Evidence from TEM and SBF SEM images indicated that that adjacent fibroblast‐like stellate cells or their processes were connected by adherent junctions (Fig. 6A,B).

TEM imaging of a fibroblast‐like stellate cell process demonstrated that regions away from the cell body contain mitochondria and rough endoplasmic reticulum (Fig. 6C).

## Discussion

This study demonstrates the presence of extracellular vesicles within the villous stroma that are much larger than any previously observed in stromal tissue or plasma. These large stromal extracellular vesicles, which we term stromal macrovesicles, were typically found in close association with fibroblast‐like stellate cells, which were themselves shown to form local networks. This suggests a higher level of organization within the stroma. These findings demonstrate how a multiscale three‐dimensional imaging approach can provide novel biological insight into features that are visible but not easily interpreted in two‐dimensional slices.

A notable finding of this study was the size of the stromal macrovesicles and the proportion of the stromal volume that they take up. In other tissues, stromal extracellular vesicles play important roles but these have volumes hundreds of times smaller than the villous stromal macrovesicles reported here (Rilla et al. 2017; Stik et al. 2017). The stromal macrovesicles reported here were surrounded by a lipid bilayer, contained little or no electron‐dense material, were typically ovoid in shape when visualized in three‐dimensions and were often in contact with a fibroblast‐like stellate cell. Although these stromal macrovesicles are easy to visualize on TEM sections, without the three‐dimensional information provided by SBF SEM they are difficult to interpret and this may explain why they have not been more extensively discussed in the literature. We are only aware of one previous study which identified similar structures in two‐dimensional images and associated these with blebs coming from pericytes (Jones & Desoye, 2011). In the current study, the stromal macrovesicles were primarily observed to be associated with fibroblast‐like stellate cells rather than pericytes and it is not clear what underlies this difference. However, in a limited number of cases a cellular process was seen extending from a pericyte or an endothelial cell to make contact with the stromal macrovesicle. It is interesting that a process extended from these cells to the stromal macrovesicle as this would be consistent with the vesicles containing a chemotrophic agent. Vesicles were also observed lying in contact with the trophoblast basal lamina, although it was not possible to discern any interaction. Given the size and abundance of the stromal macrovesicles, identifying their function is of significant interest.

The sizes of stromal vesicles in other tissues are typically reported to be in the exosome/small microvesicle size range and vesicles in this size could also be identified in the villous stroma, although not by the systematic sampling approach. The sizes of typical stromal vesicles (e.g. those in the exosome/small microvesicle size range) observed in the placenta appeared to be normally distributed and were larger than those reported in bone stromal tissue (Rilla et al. 2017). The fact that the small vesicles we observe in the villous stroma are larger than some reports in the literature may be because in three‐dimensional images the widest point can be identified, whereas two‐dimensional images may not intersect the widest point and so may under‐represent the true size of the vesicles. It was also interesting to note that, like the stromal macrovesicles, the typical stromal vesicles were not round but ovoid.

An important question is whether the typical‐size and stromal macrovesicles observed in the placenta represent one continuous population or whether they are distinct populations with distinct origins and biological roles. The small typical stromal vesicles of the size that might be observed in other tissues were not associated with fibroblast‐like stellate cells, did not have contents, and their sizes were normally distributed, suggesting that they may be a distinct population from the stromal macrovesicles. However, without a better understanding of the stromal vesicle biogenesis or the use of specific markers, this question cannot be definitively answered by this study.

It was not clear why the stromal macrovesicles associated with fibroblast‐like stellate cell processes were bigger than those that were not. Given the overlap in sizes and similarity in appearance, it is unlikely that they are different populations, but the difference in size may represent a different stage in the vesicle life course. Alternatively, if the interactions with the processes of fibroblast‐like stellate cells are dynamic, then it is more likely that the processes touch a larger stromal macrovesicle.

Although the majority of stromal macrovesicles were in close contact with stellate cells, there was no evidence that these stromal macrovesicles were joined to the stellate cells and both appeared to have discrete membranes with a lipid bilayer. If the stromal macrovesicles have roles similar to those of the much smaller stromal vesicles in other tissues they may be acting as signposts within the stroma and stellate fibroblast‐like cells may be touching them to receive or exchange chemical messages. Alternatively, the fibroblast‐like stellate cells may be secreting substances into the stromal macrovesicles or even guiding them through the stroma.

The origin of the stromal macrovesicles is not clear. Due to their size, it is unlikely that they form like exosomes within the cell or by membrane budding, like microvesicles. They are closely associated with fibroblast‐like stellate cells, but there was no evidence of them being joined to or coming from these cells. While they contain occasional membranous structures or strands of material, they do not contain organelle‐like structures, suggesting that they are not apoptotic bodies. Their size raises the question of whether they were once cells, but although large, they are much smaller than cells and their limited contents also argue against this. Another possibility is that they are derived from organelles extruded from a cell, but again, their size may argue against this (Nakajima et al. 2008).

The connections observed between pericytes and stromal macrovesicles, although few in number, resemble those reported previously connecting pericytes and large membrane‐bound vesicles in the stroma (Jones & Desoye, 2011). One interpretation of these observations is that macrovesicles bud off from pericytes and then move through the stroma, coming in contact with obstructing cell processes or membranes as they move. If this were the case, it might provide a mechanism by which vascular cells communicate with the stromal cells maintaining the local extracellular matrix.

Given the extensive presence of thin stellate processes in the stroma, another hypothesis could be that the stromal macrovesicles could form from detached processes of fibroblast‐like stellate cells. The processes of stellate cells contain organelles, so if the vesicles are derived from detached stellate processes there must be some mechanism to degrade the contents. Breakdown of the cytoplasmic contents could increase osmolarity and lead to swelling, resulting in the final shape of the vesicles. In isolated cases, stromal macrovesicles with more extensive contents were observed, which could be consistent with partially degraded organelles. However, given the evidence currently available, it is not possible to draw conclusions at this point.

What the stromal macrovesicles contain is not clear and answering this question is key to determining their function. If the stromal macrovesicles are like typical stromal vesicles, they may contain hormones and microRNA. Alternatively, they may be storage or transport vesicles. Their low electron density would be consistent with fluid‐filled vesicles. As the stromal macrovesicles have a lipid bilayer this argues against them containing lipids, because lipid droplets have a phospholipid monolayer and because lipid is usually electron dense when stained with osmium and the vesicle contents were clear (Vanni, 2017). Glycogen typically has a granular appearance on the electron microscope so it is not likely to make up the content of the vesicles (Revel et al. 1960). They could contain fluid, but it is not clear what role fluid‐filled vesicles would perform within a fluid‐filled medium, although they could play a mechanical role within the villi.

Purifying stromal macrovesicles from homogenized placental tissue will require specific markers as it would not be possible to distinguish them from vesicles formed from cell contents. Specific protein markers could be identified utilizing immunogold on TEM sections. This would allow flow cytometry of placental homogenates in order to identify and purify these stromal macrovesicles (Welsh et al. 2019). Raman spectroscopy would be an *in situ* way of identifying whether the stromal macrovesicles contain lipid (Devitt et al. 2018). Mass spectroscopy‐based microscopy may be another approach but it does not currently have the required resolution (Norris & Caprioli, 2013).

The stellate fibroblast‐like cells formed small local networks, which may allow cell–cell communication and potentially regional co‐ordination of their activities. Connections between fibroblast‐like stellate cells were apparent on the confocal and electron microscope images. The observation of junctional complexes between adjacent stellate cells suggests that these connections are more than transient contacts. The average fibroblast‐like stellate cell network as observed by confocal microscopy was comprised of just three cells; however, this may underestimate the true extent of these networks, as confocal microscopy may not effectively visualize all the thin stellate processes connecting these cells (some processes were shown to be just 0.2 μm thick by electron microscopy). As such we can demonstrate that networks exist but we cannot be sure of their extent.

The structure of the stromal stellate fibroblast‐like cells observed by SBF SEM was complicated with processes extending throughout the stroma and forming contacts with many other stromal features. As can be seen from the three‐dimensional reconstruction, fibroblast‐like stellate cell structure followed the contours of the capillary without making direct contact, while making direct connections with other fibroblast‐like stellate cells, stromal macrovesicles, and the extracellular matrix.

The three‐dimensional ultrastructural characterization of the villous stroma in healthy pregnancy presented here demonstrates how these approaches can enhance understanding of the stroma. These techniques can now be used to understand whether these structures change in disease and whether they contribute to pregnancy pathology. If the structure of the villous stroma does not effectively facilitate diffusion of nutrients and wastes, then the stroma itself may become rate‐limiting to placental function. Fetal development builds the foundation for lifelong health and the role of the stroma in this remains to be fully determined.

This study demonstrates for the first time the three‐dimensional structure of extracellular stromal macrovesicles within the villous stroma as well as their three‐dimensional interactions with fibroblast‐like stellate cells. The networks formed by fibroblast‐like stellate cells and their interaction with stromal macrovesicles suggest a higher order of organization and could form a significant barrier to diffusion across the villous stroma. These findings demonstrate the complexity of the villous stroma and the need to investigate further its role in supporting placental structure.

## Conflicts of interest

The authors have no conflicts of interest.

## Authors’ contributions

E. Palaiologou: Methodology, investigation, formal analysis, visualization, writing – original draft, writing – review & editing. O. Etter: formal analysis, investigation, visualization, writing – review & editing. P. Goggin: methodology, investigation, supervision, writing – review & editing. D. D. Chatelet: methodology, formal analysis, visualization, writing – review & editing. D. A. Johnston: methodology, investigation, writing – review & editing. E. M. Lofthouse: investigation, resources, writing – review & editing. R. Doherty: investigation, supervision, writing – review & editing. J. Pearson‐Farr: methodology, investigation, writing – review & editing. B. G. Sengers: conceptualization, writing – review & editing, funding acquisition. C. Torrens: conceptualization, writing – review & editing, funding acquisition. J. K. Cleal: conceptualization, writing – review & editing, funding acquisition. A. M. Page: methodology, conceptualization, resources, writing – review & editing, funding acquisition. R. M. Lewis: conceptualization, methodology, formal analysis, writing – original draft, writing – review & editing, funding acquisition.

## Supporting information


**Figure S1:** Interactive three‐dimensional model of stromal fibroblast‐like stellate cell and surrounding stromal macrovesicles. Note that there is an apparent break in the segmentation fibroblast‐like stellate cell caused by a slight offset in the stack.
**Figure S2:** Additional staining of fibroblasts. 1A: Confocal image of term placental villusstained with SLC22A11 in red, CD163 (macrophages) in blue and AAL (capillaries) in green. 1B shows a projection of a confocal stack showing the fibroblast‐like stellate cells stained with vimentin, a recognised fibroblast marker. The two cells on the right appear joined by their processes as seen with SCL22A11 and in the SBF SEM stacks.
