# Supplementary material for: Human placental villi contain stromal macrovesicles associated with networks of stellate cells
Source: J Anat. 2019 Sep 11;236(1):132–41. doi: 10.1111/joa.13082 (PMC6904625; doi:10.1111/joa.13082)
Supplement: Supplementary file 1 — Figure S1: Interactive three‐dimensional model of stromal fibroblast‐like stellate cell and surrounding stromal macrovesicles. Note that there is an apparent break in the segmentation fibroblast‐like stellate cell caused by a slight offset in the stack. Figure S2: Additional staining of fibroblasts. 1A: Confocal image of term placental villusstained with SLC22A11 in red, CD163 (macrophages) in blue and AAL (capillaries) in green. 1B shows a projection of a confocal stack showing the fibroblast‐like stellate cells stained with vimentin, a recognised fibroblast marker. The two cells on the right appear joined by their processes as seen with SCL22A11 and in the SBF SEM stacks. [file JOA-236-132-s001.docx]

**Supplementary Figures**

**See additional content for Supplementary Figure 1**: Interactive three-dimensional model of stromal fibroblast-like stellate cell and surrounding stromal macrovesicles. Note that there is an apparent break in the segmentation fibroblast-like stellate cell caused by a slight offset in the stack.

Supplementary figure 2

2A


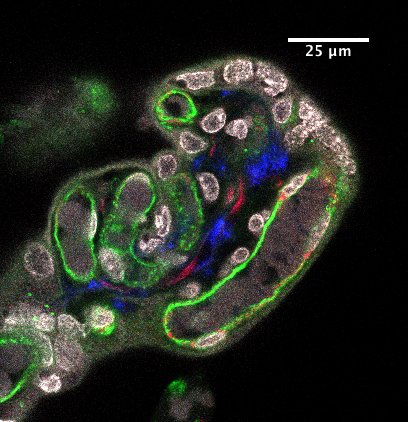


2B


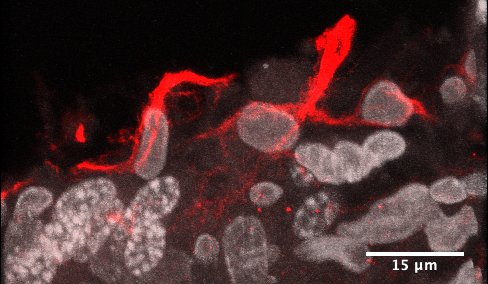


**Supplementary Figure 2**, Additional staining of fibroblasts. 1A: Confocal image of term placental villus stained with SLC22A11 in red, CD163 (macrophages) in blue and AAL (capillaries) in green. 1B shows a projection of a confocal stack showing the fibroblast like stellate cells stained with vimentin, a recognised fibroblast marker. The two cells on the right appear joined by their processes as seen with SCL22A11 and in the SBF SEM stacks.

# SBF SEM TISSUE PROCESSING SCHEDULE - High contrast protocol for serial block-face SEM

| 3% glutaraldehyde in 0.1 M cacodylate buffer at pH 7.4 | > 1 hr |
| --- | --- |
| 0.1 M cacodylate buffer rinse | 10 min |
| 0.1 M cacodylate buffer rinse | 10 min |
| Osmium/Ferrocyanide fixative | 1 hr on ice |
| *Make up Thiocarbohydrazide solution at 60˚C, agitate every 10 min* |  |
| Rinse in distilled water | 5 x 3 min |
| Thiocarbohydrazide solution (made 1 hr before use) at RT. | 20 min |
| Distilled water rinse | 5 x 3 min |
| 2% Osmium Tetroxide | 30 min |
| Distilled water rinse | 5 x 3 min |
| 2% Uranyl acetate at 4˚C | 1 hr |
| *Make up Walton’s lead aspartate solution at 60˚C* |  |
| Distilled water rinse | 5 x 3 min |
| Walton’s lead aspartate solution at 60˚C (make up 30 min before use) | 30 min |
| Distilled water rinse | 5 x 3 min |
| 30% ethanol | 10 min |
| 50% ethanol | 10 min |
| 70% ethanol | 10 min |
| 95% ethanol | 10 min |
| Absolute ethanol | 20 min |
| Absolute ethanol | 20 min |
| Acetonitrile | 20 min |
| 50:50 Acetonitrile:Spurr resin | Overnight |
| Spurr resin | 6 hr |
| Embed in fresh resin and polymerise at 60˚C | 16 hr |

**Osmium/Ferrocyanide fixative**

| 3% potassium ferrocyanide in 0.3 M cacodylate buffer plus 4 mM calcium chloride (0.15 g in 5mls buffer) | 5 ml |
| --- | --- |
| 4% osmium tetroxide | 5 ml |

Method

1. Mix the 2 components together just before use to produce 1.5% potassium ferrocyanide plus 2% osmium tetroxide in 0.15 M cacodylate buffer plus 2 mM calcium chloride, pH 7.4.

**1% Thiocarbohydrazide solution (make up 1 hr prior to use)**

| Thiocarbohydrazide | 0.1 g |
| --- | --- |
| Distilled water | 10 ml |

Method

1. Mix the 2 components together and place in an oven at 60˚C for 1 hr (agitate by swirling every 10 min).
2. Filter through 0.22 um Millipore filter before use.

**Walton’s lead aspartate solution**

| Lead nitrate | 0.066 g |
| --- | --- |
| 0.03 M aspartic acid | 10 ml |

Method

1. Mix the 2 components together and adjust to pH 5.5 with 1 M KOH.
2. Place in oven for 30 min (no precipitate should form).
